# Monitoring hearing and vision functions in older adults: rationale and process

**DOI:** 10.1093/ageing/afad123

**Published:** 2023-10-30

**Authors:** Shelly Chadha, Lauren K Dillard, Silvio P Mariotti, Stuart Keel

**Affiliations:** Department on Noncommunicable Diseases, World Health Organization, Geneva, Switzerland; Department of Otolaryngology—Head & Neck Surgery, Medical University of South Carolina, Charleston, SC, USA; Department on Noncommunicable Diseases, World Health Organization, Geneva, Switzerland; Department on Noncommunicable Diseases, World Health Organization, Geneva, Switzerland

**Keywords:** hearing loss, blindness, vision screening, community health services, monitoring, older people

## Abstract

Hearing and vision impairment are highly prevalent in ageing individuals and are significant public health concerns given their meaningful impacts on individuals and society. Yet, many cases of both visual and hearing impairment remain unidentified and thus, unaddressed. This article describes the rationale and process of monitoring for visual and hearing impairment in older adults, by summarising guidance and resources available from the World Health Organisation (WHO) that were developed based upon the best current available evidence. It is recommended that vision screening be offered at least annually to adults aged over 50 years and hearing screening be offered every 5 years to adults aged 50–64 years, and every 1–3 years to adults aged 65 years or older. Both hearing and vision screening can be conducted in community, home or clinical settings by trained health workers with simple equipment. More specifically, vision screening can be conducted with a simple eye chart. Hearing screening can be conducted without specialised equipment by using pure tones set to a fixed level, an automated mobile- or web-based digits-in-noise test, or the whispered voice test. Hearing screening can also be conducted in audiology clinics using pure-tone air conduction threshold testing. There exists WHO guidance to support the monitoring of hearing and vision impairment, which, when warranted, can facilitate referral for comprehensive assessment and prompt appropriate, person-centred interventions to mitigate the negative consequences of hearing and vision impairment.

## Key Points

- The World Health Organisation has guidance to support the monitoring of hearing and vision impairment.
- Hearing and vision screening can be conducted in community, home or clinical settings.
- Hearing and vision screening can be performed by trained health workers with simple equipment.
- Monitoring for hearing and vision impairment can prompt appropriate, person-centred interventions.

## Importance of regular monitoring of hearing and vision

The human body interacts with the external environment with the use of its five special senses: hearing, vision, smell, touch and taste [1]. The loss or impairment of any of these senses can have an adverse impact on the quality of life of affected individuals. Of these, the two that are most common and have been highlighted as significant public health concerns are impairments in vision or hearing [2–4].

Globally, it is estimated over 2.2 billion people have a vision impairment or blindness, whilst over 1.5 billion live with hearing impairment, the majority of whom are over 60 years of age [3, 4]. The prevalence of these sensory dysfunctions rises with age and in adults above 70 years of age, hearing loss and vision impairment are the leading and third-leading causes of Years Lived with Disability, respectively [5]. Furthermore, an estimated 0.2–2.0% of the global population have impairment of both hearing and vision [3, 6]. The prevalence and burden of hearing and vision impairments are estimated to rise drastically as the global population ages, highlighting the urgent need to monitor these sensory impairments, which will facilitate their management [3, 4].

When hearing and vision impairments remain unaddressed, they pose a significant challenge for the quality of life of those affected [2–4]. Hearing and vision impairment are independently and collectively associated with poorer quality of life and negative functional impacts on physical and mental health [2]. Furthermore, hearing and vision impairment are associated with cognitive decline [7–9], and hearing loss has been identified as the most common modifiable risk factor for dementia in older adults [10]. Their impacts are also felt at a larger societal level. Conservative estimates suggest the annual global productivity loss from vision impairment is ⁓410.7 billion US dollars, and unaddressed hearing loss alone poses an annual global cost of nearly 1 trillion US dollars [11, 12].

For many people (over 800 million) with refractive errors, vision impairment could simply be addressed with an appropriate pair of spectacles, and for others corrected through cataract surgery, and over 400 million of those with hearing loss could benefit through use of a hearing aid [3, 4]. Despite this, neither eye care, vision screening nor hearing screening or services are commonly integrated within health systems, and the vast majority of those in need of spectacles (an estimated 64%) and hearing aids (an estimated 83%) do not have access to them [3, 4, 13, 14]. Many adults living in low- and middle-income countries have never had an eye examination or a hearing test and currently do not have the option to access these services [3, 4].

Challenges in managing hearing and vision impairment are further aggravated by the lack of systematic monitoring for vision and hearing capacities in older adults [3, 4]. As a result, it is estimated that people commonly wait for many years to seek a vision or hearing test and to receive the appropriate treatment [3, 15].

In consideration of the above-mentioned facts, the World Health Organisation (WHO) ‘Integrated care for older people: guidelines on community-level interventions to manage declines in intrinsic capacity’ published in 2017 recommended the integration of systematic and routine vision and hearing screening followed by provision of appropriate care and rehabilitation for older adults as being essential to suitably manage the declines in physical and mental capacities [16–18]. Moreover, it is important to monitor and report on hearing capacity of older adults and access to services as part of determining health system requirements and monitoring service provision for health of older adults. There exists detailed guidance, as well as a mobile application (WHO ICOPE App), to aid in the implementation and monitoring of the integrated care for older people framework, which includes hearing and vision screening [16, 17, 19].

WHO publications including the World report on vision, Package of eye care interventions, World report on hearing, Hearing screening: considerations for implementation, and the soon-to-be published WHO Sensory function survey methodology, provide guidance on how hearing and vision assessment can be routinely undertaken [3, 4, 20, 21].

This guidance is based on the best available evidence collated by WHO. Importantly, it demonstrates how both hearing and vision screening can be conducted with simple equipment in clinical, community or home settings by trained health workers. Wherever feasible, hearing and vision screening should be aligned with other health checks offered to older adults and screening should be embedded in health systems. The steps for hearing and vision screening are summarised below.

### Eye examination and vision screening

These services should be offered at least once a year to adults over the age of 50 years [16]. Eye examination is recommended every 5 years in the absence of signs or symptoms of changes in function. In the case of any systemic disease (e.g. diabetes, hypertension) or treatments with an impact on eye health (e.g. corticosteroids) comprehensive eye examinations are recommended more frequently according to the severity of the disease or the frequency and duration of treatments. Vision function screening can be performed at community or primary health system settings by trained personnel, whilst comprehensive eye examination shall be performed by well-trained eye care professionals.

All people are requested to share their perceived vision function status and ongoing diseases or treatment they are aware of. In addition, all people undergo a distance and near vision testing. This is performed with distance and near optotypes, wearing spectacles if usually worn by the person. Vision apps (such as WHOEyes App) could also be used. All vision testing shall be performed with adequate lighting and correct distance by trained screeners.

Any fails should be referred for refractive assessment and care. In the case of reported diseases or treatment, or if reporting having undergone eye examination ˃5 years before (or never), individuals should be referred to undergo comprehensive eye examination by a trained eye professional.

### Hearing screening

It is recommended that screening be offered to all adults above the age of 50 years and may be conducted at 5-yearly intervals until the age of 64 years. From 65 years of age, the frequency of screening should be increased to every 1–3 years. Hearing screening can be conducted by trained health workers and nurses, community health workers, non-hearing care clinicians or ear and hearing care clinicians, and with one of the four following simple tests [16, 21]. (i) Automated screeners set to detect 35 dB can detect moderate or higher severity of hearing loss. The level can be defined by the detection of pure tones in each ear, separately, at a fixed decibel level at frequencies 1.0, 2.0 and 4.0 kHz. (ii) The automated digits-in-noise test can also be used to screen for hearing loss, in which case both ears are tested together. Several mobile applications (e.g. hearWHO) or web-based services provide access to an automated digits-in-noise test [21]. (iii) The whispered voice test should be the preferred option where none of the above-mentioned options for screening are available. The prior three methods are suitable for community-based or clinical settings other than an audiology clinic. (iv) Detection of pure-tone air conduction thresholds at frequencies 0.5, 1.0, 2.0 and 4.0 kHz. This option is most suitable when applied in audiology clinics to reduce ambient noise that may be more common in other settings. For hearing screening that is conducted in community or clinical (not audiology clinic) settings, care must be taken to ensure background noise levels below 40 dB.

Those who fail a hearing screening should be referred to a clinician for diagnostic assessment, which can be done by pure-tone audiometry [16]. Individuals who pass the screening should be counselled on basic ear and hearing care, advised to undergo repeat screening at a specified future time point (1–5 years, dependent on age) and to seek medical care in the event of developing any symptom.

### Managing hearing and vision impairment

For individuals diagnosed with vision or hearing impairment, it is essential that the impairment is addressed as early as possible and in an appropriate manner to mitigate any adverse impacts. Thus, intervention strategies must be planned and available at the time of screening. These intervention strategies must be person-centred and take into account the individual’s needs and preferences, in addition to the available resources [16, 21]. Interventions for hearing and vision impairment should incorporate basic education and counselling to facilitate psychological acceptance of and adjustment to the impairment. This counselling should include information on modifications that reduce negative impacts of impairment, such as improving lighting to improve visibility and supporting communication strategies to mitigate impacts of hearing impairment [16]. For vision impairment, intervention includes management of eye diseases (e.g. cataract surgery) or recommendations to receive spectacles or other vision rehabilitation interventions including assistive devices [16, 20]. For hearing impairment, interventions may include recommendations for hearing aid use or other forms of aural rehabilitation such as lip-reading or other communication strategies.

## Conclusions

Screening likely supports the early identification of and intervention for hearing and vision impairment. Both hearing and vision screening can be conducted in clinical, community or home settings by trained health workers with simple equipment. The WHO has published guidance that outlines the process of monitoring for hearing and vision impairment. When warranted, this monitoring can facilitate prompt, integrated, person-centred interventions that mitigate the negative consequences of hearing and vision impairment.

## Data Availability

No new data was generated or analysed for this article.
